# Supplementary figures and images for: Phosphorylation of tyrosine 90 in SH3 domain is a new regulatory switch controlling Src kinase
Source: eLife. 2023 Jul 10;12:e82428. doi: 10.7554/eLife.82428 (PMC10361714; doi:10.7554/eLife.82428)

Figure 1A

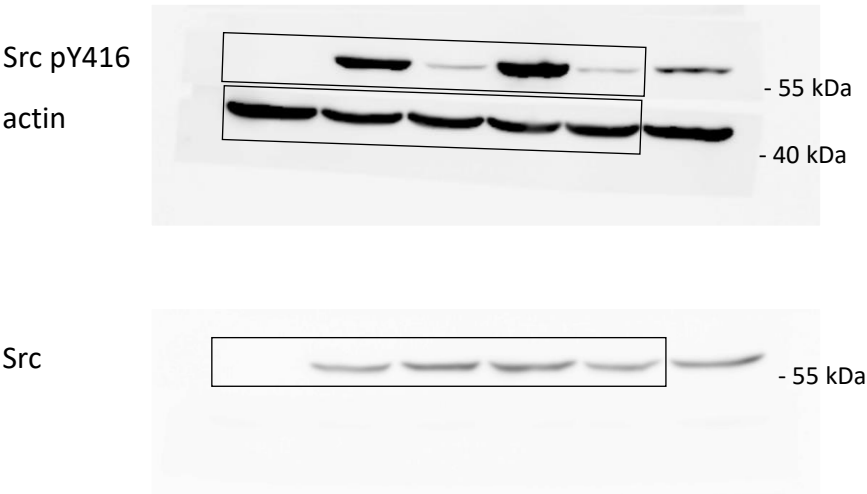

Supplement: Figure 1—source data 1. [file elife-82428-fig1-data1.zip › Figure 1 source data/Figure 1 blots.pdf]

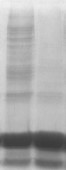

Supplement: Figure 2—source data 1. [file elife-82428-fig2-data1.zip › Figure 2 source data/Fig 2A blot.jpg]

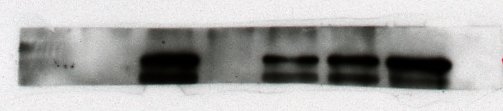

Supplement: Figure 2—source data 1. [file elife-82428-fig2-data1.zip › Figure 2 source data/Fig 2B blot Cas.jpg]

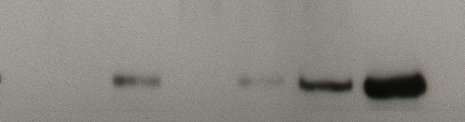

Supplement: Figure 2—source data 1. [file elife-82428-fig2-data1.zip › Figure 2 source data/Fig 2B blot FAK.jpg]

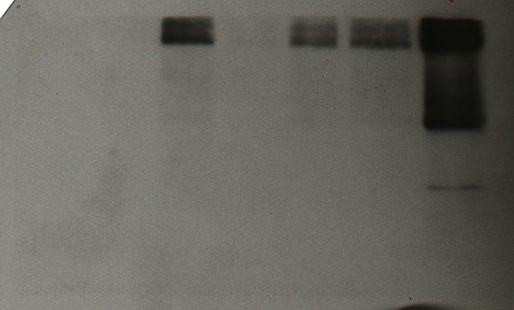

Supplement: Figure 2—source data 1. [file elife-82428-fig2-data1.zip › Figure 2 source data/Fig 2B blot paxillin.jpg]

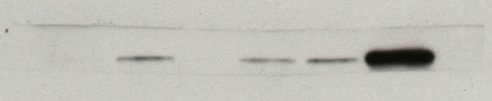

Supplement: Figure 2—source data 1. [file elife-82428-fig2-data1.zip › Figure 2 source data/Fig 2B blot Stat3.jpg]

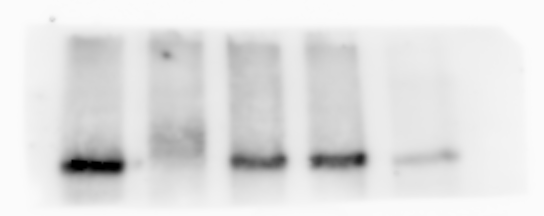

Supplement: Figure 2—source data 1. [file elife-82428-fig2-data1.zip › Figure 2 source data/Fig 2C blot IP Cas.tif]

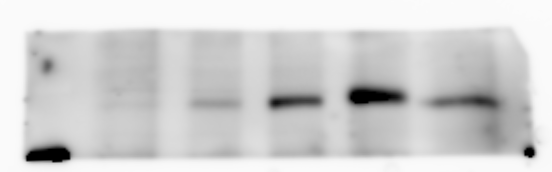

Supplement: Figure 2—source data 1. [file elife-82428-fig2-data1.zip › Figure 2 source data/Fig 2C blot IP FAK.tif]

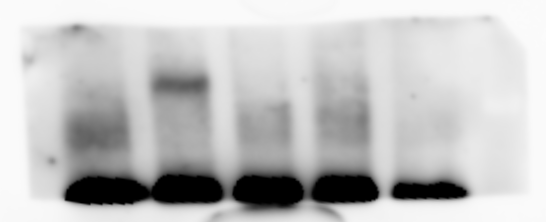

Supplement: Figure 2—source data 1. [file elife-82428-fig2-data1.zip › Figure 2 source data/Fig 2C blot IP paxillin.tif]

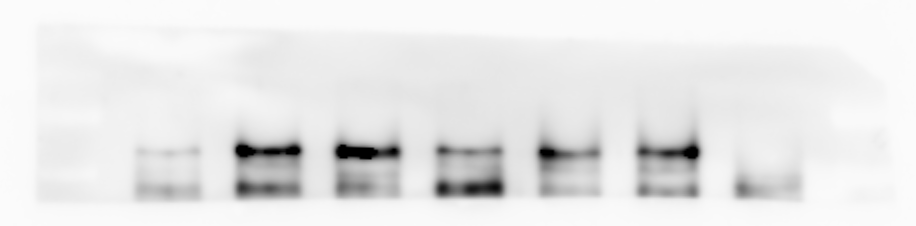

Supplement: Figure 2—source data 1. [file elife-82428-fig2-data1.zip › Figure 2 source data/Fig 2C blot IP Src.tif]

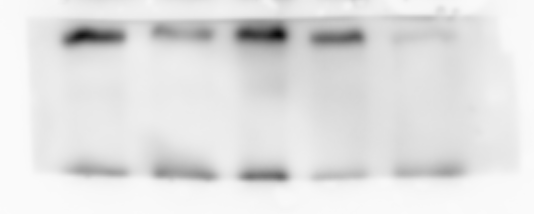

Supplement: Figure 2—source data 1. [file elife-82428-fig2-data1.zip › Figure 2 source data/Fig 2C blot IP Stat3.tif]

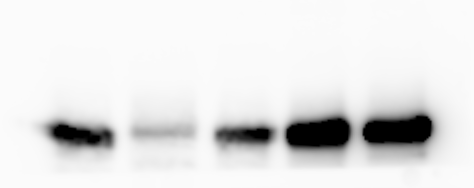

Supplement: Figure 2—source data 1. [file elife-82428-fig2-data1.zip › Figure 2 source data/Fig 2C blot TL Cas.tif]

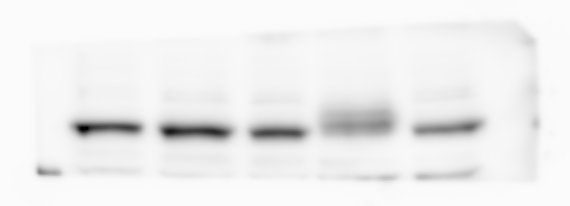

Supplement: Figure 2—source data 1. [file elife-82428-fig2-data1.zip › Figure 2 source data/Fig 2C blot TL FAK.tif]

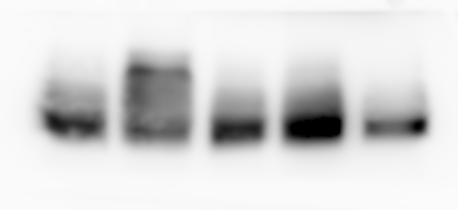

Supplement: Figure 2—source data 1. [file elife-82428-fig2-data1.zip › Figure 2 source data/Fig 2C blot TL paxillin.tif]

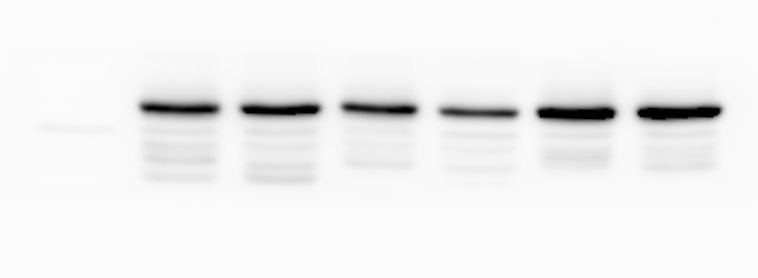

Supplement: Figure 2—source data 1. [file elife-82428-fig2-data1.zip › Figure 2 source data/Fig 2C blot TL Src.tif]

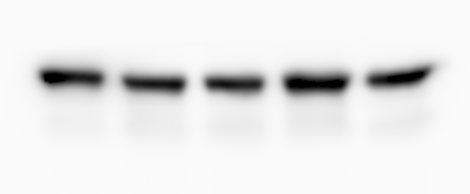

Supplement: Figure 2—source data 1. [file elife-82428-fig2-data1.zip › Figure 2 source data/Fig 2C blot TL Stat3.tif]

Figure 5D

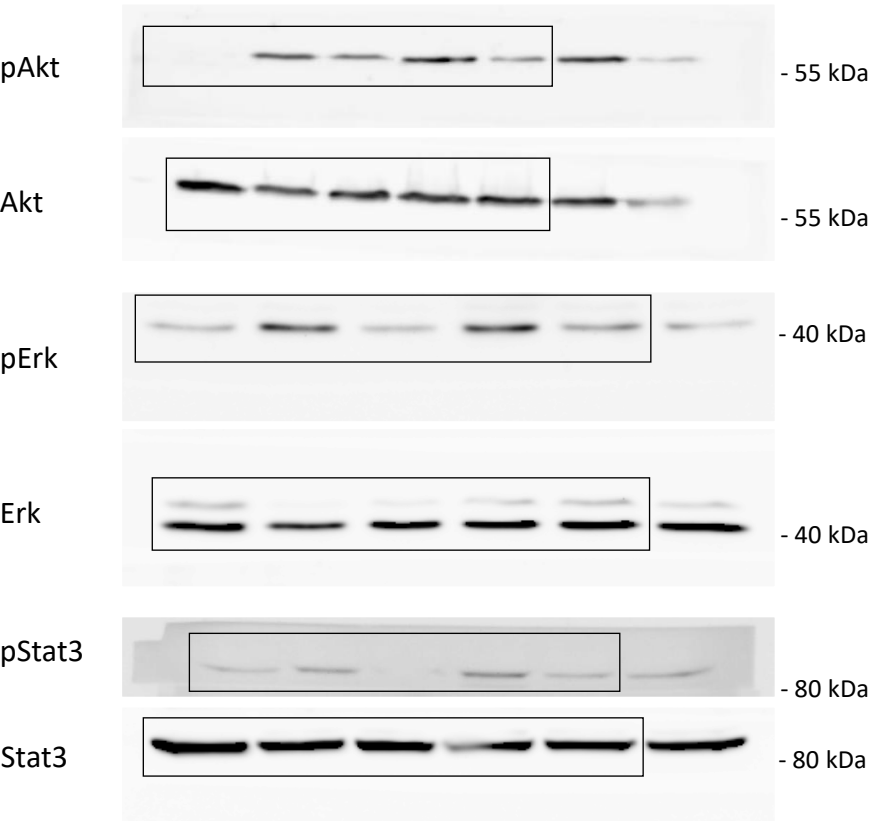

Supplement: Figure 5—source data 1. [file elife-82428-fig5-data1.zip › Figure 5 source data/Figure 5 blots.pdf]

**Figure 6D**

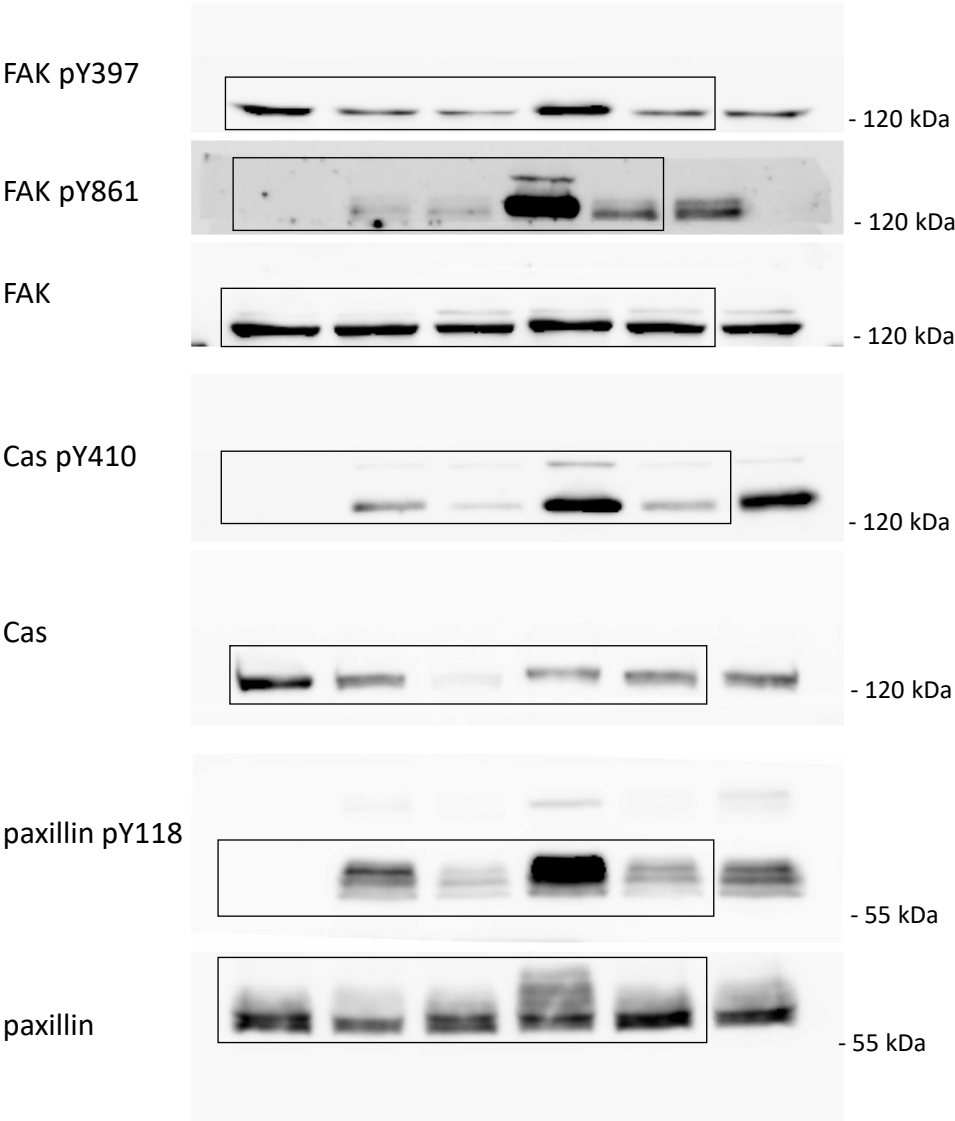

Supplement: Figure 6—source data 1. [file elife-82428-fig6-data1.zip › Figure 6 source data/Figure 6 blots.pdf]
