# Supplementary material for: Phosphorylation of tyrosine 90 in SH3 domain is a new regulatory switch controlling Src kinase
Source: eLife. 2023 Jul 10;12:e82428. doi: 10.7554/eLife.82428 (PMC10361714; doi:10.7554/eLife.82428)
Supplement: Figure 2—source data 1. [file elife-82428-fig2-data1.zip › Figure 2 source data/Figure 2 blots.pdf]

Figure 2A

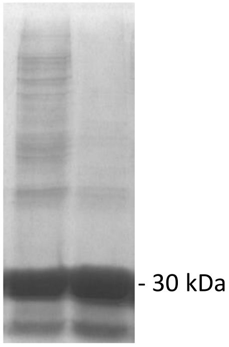

Figure 2B

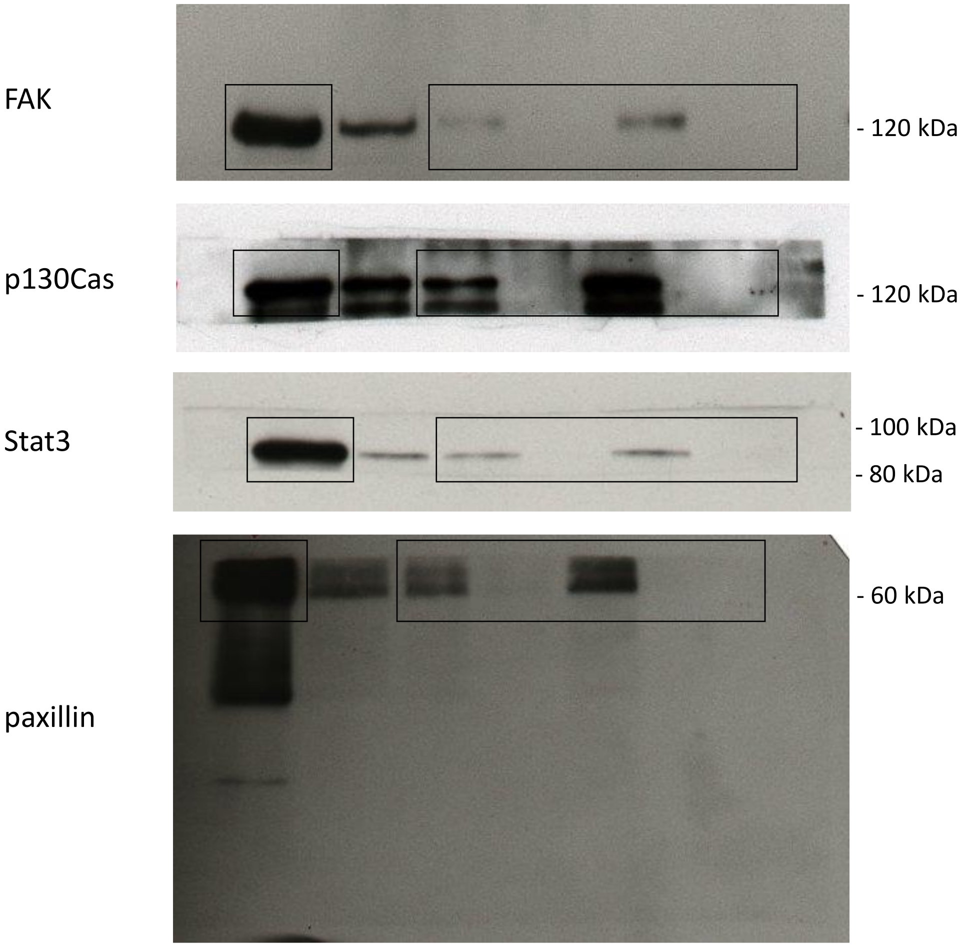

Figure 2C

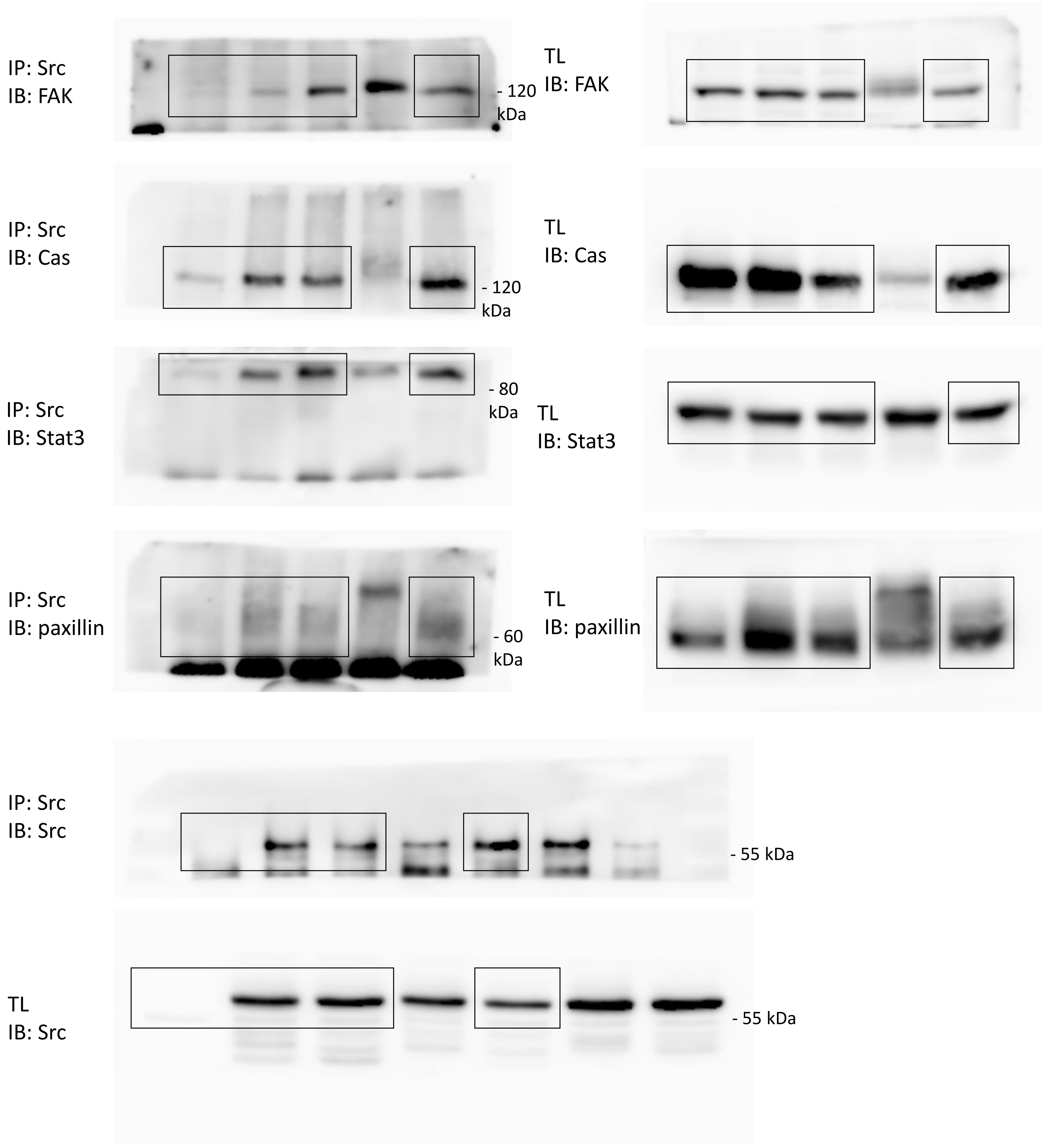
IP: Src
IB: FAK
- 120 kDa
TL
IB: FAK
IP: Src
IB: Cas
- 120 kDa
TL
IB: Cas
IP: Src
IB: Stat3
- 80 kDa
TL
IB: Stat3
IP: Src
IB: paxillin
- 60 kDa
TL
IB: paxillin
IP: Src
IB: Src
- 55 kDa
TL
IB: Src
- 55 kDa
